# Existence of the *rdl *mutant alleles among the *anopheles *malaria vector in Indonesia

**DOI:** 10.1186/1475-2875-11-57

**Published:** 2012-02-25

**Authors:** Puji BS Asih, Lepa Syahrani, Ismail EP Rozi, Nandha R Pratama, Sylvia S Marantina, Dian S Arsyad, Wibowo Mangunwardoyo, William Hawley, Ferdinand Laihad, Shinta, Supratman Sukowati, Neil F Lobo, Din Syafruddin

**Affiliations:** 1 Eijkman Institute for Molecular Biology, Jalan Diponegoro, 69, Jakarta 10430, Indonesia; 2 Department of Biology, Faculty of Mathematic and Science, University of Indonesia, Depok 16424, Indonesia; 3 Department of Parasitology, Faculty of Medicine, Hasanuddin University, Makassar 90245, Indonesia; 4 UNICEF, Jakarta, 12920, Indonesia; 5 Center for Communicable Diseases Control and Prevention (CDC), Atlanta, USA; 6 Health Ecology Research & Development Centre, National Institute of Health, Research and Development, Department of Health, Jalan Percetakan Negara 29, Jakarta, 10560, Indonesia; 7 Eck Institute for Global Health, University of Notre Dame, Notre Dame, IN, USA

**Keywords:** *Anopheles*, Diedrin, GABA, Receptor, Malaria, *Rdl*

## Abstract

**Background:**

The gamma-aminobutyric acid (GABA) receptor-chloride channel complex is known to be the target site of dieldrin, a cyclodiene insecticide. GABA-receptors, with a naturally occurring amino acid substitution, A302S/G in the putative ion-channel lining region, confer resistance to cyclodiene insecticides that includes aldrin, chlordane, dieldrin, heptachlor, endrin and endosulphan.

**Methods:**

A total of 154 mosquito samples from 10 provinces of malaria-endemic areas across Indonesia (Aceh, North Sumatra, Bangka Belitung, Lampung, Central Java, East Nusa Tenggara, West Nusa Tenggara, West Sulawesi, Molucca and North Molucca) were obtained and identified by species, using morphological characteristic. The DNA was individually extracted using chelex-ion exchanger and the DNA obtained was used for analyses using sequencing method.

**Results:**

Molecular analysis indicated 11% of the total 154 *Anopheles *samples examined, carried *Rdl *mutant alleles. All of the alleles were found in homozygous form. *Rdl *302S allele was observed in *Anopheles vagus *(from Central Java, Lampung, and West Nusa Tenggara), *Anopheles aconitus *(from Central Java), *Anopheles barbirostris *(from Central Java and Lampung), *Anopheles sundaicus *(from North Sumatra and Lampung), *Anopheles nigerrimus *(from North Sumatra), whereas the 302 G allele was only found in *Anopheles farauti *from Molucca.

**Conclusion:**

The existence of the *Rdl *mutant allele indicates that, either insecticide pressure on the *Anopheles *population in these areas might still be ongoing (though not directly associated with the malaria control programme) or that the mutant form of the *Rdl *allele is relatively stable in the absence of insecticide. Nonetheless, the finding suggests that integrated pest management is warranted in malaria-endemic areas where insecticides are widely used for other purposes.

## Background

Malaria parasites in Indonesia are transmitted by 24 species of *Anopheles *mosquitoes [1] that vary markedly in biological attributes, including patterns of blood feeding, response to volatile insecticides, and larval habitats. Such variation will impact the effectiveness of insecticide-treated nets (ITNs), indoor residual spraying (IRS) and larval habitat treatments or modifications [2]. Malaria control strategies in Indonesia are aimed at the *Anopheles *malaria vector and rapid treatment of patients [3,4]. Control of malaria vectors has been done using insecticides that target the immatures and adult stage [5,6].

Vector control uses a group of organochlorine insecticides, organophosphates, pyrethroids, and carbamates to kill mosquitoes [7]. However, continuous use of insecticides at high frequency and over long periods without inadequate supervision selects for resistant strains of mosquitoes. This resistance causes a decrease in target susceptibility in the mosquito population with a reduction in the efficacy of the vector control programme. Currently, a total 125 species of mosquitoes, including the genus *Anopheles *have been recorded to be resistant to one or more insecticides [8].

Organochlorine insecticides are classified into three groups; dichlororodiphenyl trichlor ethane (DDT), hexachlorhexana (HCH) and cyclodiene (aldrin, chlordane, heptachlor, dieldrin, endrin and endosulfan) [9]. DDT was used in malaria eradication programmes in Indonesia in the early 1950s but was subsequently banned in the 1970s as resistance to DDT emerged and spread rapidly. Dieldrin (cyclodiene) was introduced to malaria control programme in Indonesia since 1955 [10,11]. The use of dieldrin in health programmes proved to be highly effective and its use was promoted in agriculture [12]. The rapid development of mosquito resistance to this insecticide later prompted the national malaria control program to terminate the use of dieldrin in 1965. However, several cyclodiene compounds, such as endosulfan and endrin are currently still used as pesticide in Indonesia.

Mosquito resistance to insecticides has been detected in recent years following insecticide use. Some species of *Anopheles *have demonstrated resistance to dieldrin. *Anopheles albimanus *in El Salvador, *Anopheles gambiae *[13] and *Anopheles sacharovi *in Turkey [14] have shown resistance to DDT and dieldrin [15]. Khan (1961) reported multiple resistance to dieldrin and DDT in *Aedes aegypti *in Puerto Rico [16]. In Indonesia, double resistance to DDT and dieldrin has been reported through biochemical tests of *Anopheles aconitus *in Central Java [17].

Earlier, it was demonstrated that resistant traits depend on major genetic factors and that the nervous system of the resistant insect is more tolerant to the action of cyclodienes [18]. Ghiasuddin and Matsumura (1982) [19] first proposed that the GABA receptor is the target of these cyclodiene insecticides and this was later confirmed by others [20-22]. Resistance to dieldrin involves a subunit of the insect gamma aminobutyric acid (GABA) receptor, The encoded *Rdl *subunit assembles with other GABA receptor subunits to form the target site of the cyclodiene insecticides [23]. Dieldrin resistance is associated with the replacement of a single amino acid (alanine at position 302) in the *Drosophila melanogaster Rdl *allele [24]. A homologous mutation has also been indicated to confer dieldrin resistance in a wide variety of insect species such as *A. aegypti*, *Drosophila simulans, Musca demestica, Lucilia cuprina, Blattella germanica*, *Tribolium castaneum*, *Hypothenemus hampei, Bemisia*, and *Myzus persicae*. Resistance to dieldrin has been particularly associated with the single nucleotide polymorphisms in the M2 transmembrane domain of the GABA-gated chloride ion channel (*Rdl *allele) [25,26]. The present study aims to explore the allelic distribution of the *Rdl *gene among the Anopheline malaria vectors from different malaria endemic areas of Indonesia.

## Methods

### Study area of mosquito collection

**Figure 1:**
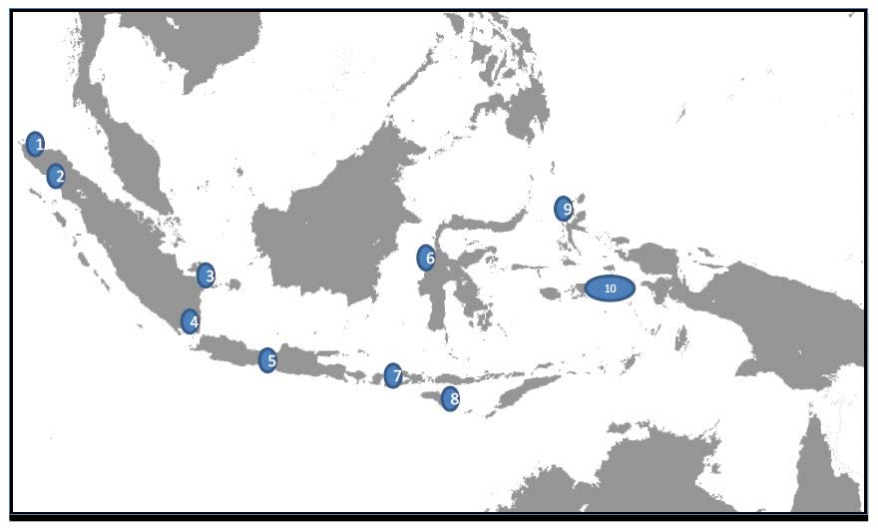
**Study area of mosquito collection**. *Anopheles *species were collected from several provinces in Indonesia: 1. Aceh 2. North Sumatra 3. Bangka Belitung 4. Lampung 5. Central Java 6. West Sulawesi 7. West Nusa Tenggara 8. East Nusa Tenggara 9. North Molucca 10. Molucca.

Female a*nopheline *mosquitoes were collected from 10 provinces across Indonesia with different malaria area endemicities - Aceh, North Sumatra, Bangka Belitung, Lampung, Central Java, East Nusa Tenggara, West Nusa Tenggara, West Sulawesi, Molucca and North Molucca (Figure 1). After morphological identification to species, mosquitoes were stored individually in a 1.5 ml Eppendorf microtube containing cotton flap and silica gel and kept at 4°C until use.

### Extraction of mosquito DNA

Mosquitoes were ground with teflon pestles in 50 μl blocking buffer (BB), containing 5.0 g Casein; 0.01 g/L Phenol Red; 900 ml phosphate buffered saline (PBS), pH 7.4; 100 ml of 0.1 N NaOH; with additional IGEPAL (5 ul IGEPAL: 1 ml BB). The teflon pestles were subsequently rinsed with additional 200 μl volume of blocking buffer. Mosquito DNA from 50 μl homogenate was extracted using chelex-100 ion exchanger (Biorad Laboratories, Hercules, CA) essentially according to the procedure described previously [27]. The remaining 200 μl homogenate was used for other analysis. The DNA was either used immediately for a polymerase chain reaction (PCR) or stored at −20°C for later analysis.

### Gene amplification with the seminested-PCR

Semi-nested PCRs were performed on the *Rdl *gene. All reactions were carried out in 25 μl reaction mixtures containing 50 mM KCl, 10 mM Tris-HCl pH 8.3, 1.5 mM MgCl2, 200 mM dNTP, 1 U Taq Polymerase and a pair of primers (20 pM each). 1-5 μl of DNA was used as template in the first reaction and 1-2 μl of the first round PCR product was used as template for the secondary PCR. Secondary PCR products were resolved by electrophoresis on 2% agarose gel and visualized by staining with ethidium bromide. The *Rdl *gene was amplified using primers RDLF F10 (5 'SAG TTT TCG ATG CGT GTA TAT GGT WW 3'), F11 RDLF (5 'AGC ATG TGA AAT TTK ASA G 3 '), and R12 RDLF (5' CCA CAA ATA GCA TGG GAC CCA RGA 3 '). The initial nucleotide S is C/G, W is T/A, K is T/G, and R is A/G. Cycling conditions for first PCR using oligos F11 × R12 RDLF was denaturation at 94°C, annealing at 50°C, extension at extension at 72°and final polymerization at 72°C, each phase lasts for 30 s, 30 s, 1 min and 30 s, and 5 min to 30 cycles. The second round PCR conditions used oligos F10 × F12 RDLF for the stages of denaturation, annealing, extension, and final polymerization are 94°C, 50°C, 72°C, and 72°C, each phase lasts for 30 s, 30 s, 45 s and 45 s (40 cycles). The final PCR products of approximately 250 bp in size were sequenced in all individual mosquitoes. The PCR products were purified using PCR clean up system (PROMEGA Corporation, Madison, WI, USA). The purified amplicons were sequenced using an ABI Prism™ Dye BigDye Terminator Cycle Sequencing Ready Kit (Applied Biosystem, Foster City, USA) in automatic sequencer fluorescent DNA capillary electrophoresis (ABI 3130 × l) at the Eijkman Institute, Jakarta, Indonesia.

## Results

### PCR amplification and DNA sequencing of the fragment of *rdl *gene of various anopheles species from Indonesia

**Figure 2:**
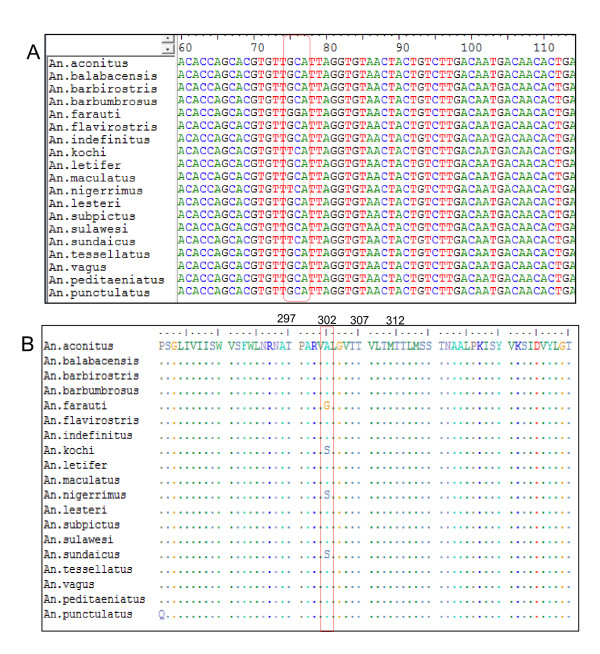
**Sequence aligment**. **a**. DNA sequence aligment of the fragment of Rdl gene in 19 various Anopheles species from Indonesia; **b**. Amino acid sequences of the GABA receptor encoded by Rdl gene encompassing the mutation site, codon A302S/G in 19 various Anopheles species from Indonesia. Numbers indicate the codon in GABA receptor.

Using primers that has been designed based on the published sequence of *Rdl *gene from *An*. *gambiae *(GenBank acc no. AF470112 and AF470116), *Anopheles stephensi *(GenBank acc no. EU883213), *Aedes aegypti *(GenBank acc no. AAU28803), and *Culex quinquefasciatus *(GenBank acc no. XM001850045) and sequences of *Anopheles sundaicus *from Indonesia (GenBank acc no. JN675907-JN675923), encompasssing the *Rdl *gene mutation site was successfully amplified and amplicons of approximately 250 bp in size in 19 A*nopheles species *were obtained. Amplicons were then sequenced and submitted to GenBank acc no. JN690008 - JN690025. Alignment of the 207 bp DNA sequencing results of each species is shown in Figure 2. The DNA sequences of the *Rdl *gene showed 12 variable nucleotide sites among the *Anopheles *species analyzed but the deduced amino acid sequences indicated a high sequence conservation.

### Existence of rdl allele

**Figure 3:**
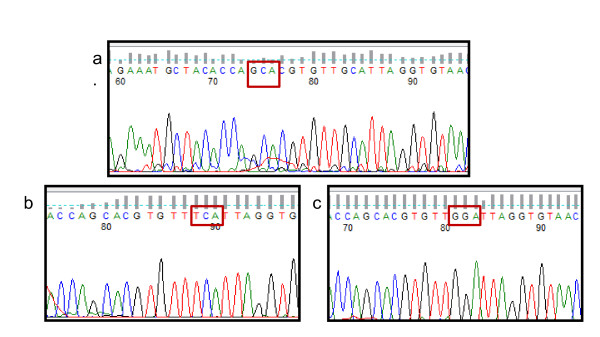
**Electropherogram of the DNA sequencing of GABA-*Rdl *gene**. a. indicated the wildtype allele and b. indicated the resistance alele GCA. (alanin) replacement by TCA (serine) and c. GCA replacement by GGA (glycine).

Analysis of DNA sequences of 154 amplicons representing 19 *Anopheles *species indicated that the majority of the *Anopheles *carried the wildtype 302A allele. The 302S polymorphism of the *Rdl *gene, popularly known as *Rdl *allele was detected in four provinces: North Sumatra, Central Java, Lampung and West Nusa Tenggara while the 302 G allele was detected in Molucca Province (Table 1). The *Rdl *allele was detected in *Anopheles vagus, An*. *aconitus, Anopheles barbirostris, Anopheles sundaicus *and *Anopheles nigerrimus *whereas the 302 G allele was only detected in *Anopheles farauti *from Molucca (Figure 3). All of the alleles were found in homozygous form. The *Rdl *302S/G allele was not found in any of the *Anopheles *species examined from Aceh, Bangka Belitung, West Sulawesi, East Nusa Tenggara and North Molucca.

**Table 1:** Frequency of *Rdl *allele in each *Anopheles *species examined at each study site

Study site Provinces	Species	N Σ = 154	Genotype frequency%			Allele frequency(%)		
			
			AA	SS	GG	A	S	G
North Molluca	*An. punctulatus*	2	100	0	0	100	0	0
	
	*An. subpictus*	1	100	0	0	100	0	0
	
	*An. tesselatus*	1	100	0	0	100	0	0
	
	*An. kochi*	3	100	0	0	100	0	0
	
	*An. barbumbrosus*	1	100	0	0	100	0	0
	
	*An. farauti*	1	100	0	0	100	0	0
	
	*An. lesteri*	1	100	0	0	100	0	0
	
	*An. vagus*	3	100	0	0	100	0	0

Molluca	*An. punctulatus*	4	100	0	0	100	0	0
	
	*An. lesteri*	1	100	0	0	100	0	0
	
	*An. farauti*	10	90	0	10	90	0	10

North Sumatra	*An. vagus*	4	100	0	0	100	0	0
	
	*An. peditaeniatus*	1	100	0	0	100	0	0
	
	*An. nigerrimus*	3	67	33	0	67	33	0
	
	*An. sundaicus*	8	87.5	12.5	0	87.5	12.5	0

Central Java	*An. vagus*	4	50	50	0	50	50	0
	
	*An. aconitus*	11	55.5	45.5	0	55.5	45.5	0
	
	*An. barbirostris*	8	75	25	0	75	25	0
	
	*An. Maculatus*	1	100	0	0	100	0	0
	
	*An. balabacensis*	6	100	0	0	100	0	0

Lampung	*An. vagus*	5	60	40	0	60	40	0
	
	*An. sundaicus*	23	96	4	0	96	4	0
	
	*An. barbirostris*	1	0	100	0	0	100	0

Bangka Belitung	*An. sundaicus*	10	100	0	0	100	0	0
	
	*An. letifer*	3	100	0	0	100	0	0

East Nusa tenggara	*An. vagus*	1	100	0	0	100	0	0
	
	*An. sundaicus*	2	100	0	0	100	0	0
	
	*An. subpictus*	4	100	0	0	100	0	0
	
	*An. flavirostris*	2	100	0	0	100	0	0
	
	*An. indefinitus*	3	100	0	0	100	0	0
	
	*An. barbirostris*	1	100	0	0	100	0	0
	
	*An. tesselatus*	1	100	0	0	100	0	0
	
	*An. kochi*	1	100	0	0	100	0	0
	
	*An. maculatus*	1	100	0	0	100	0	0

West Nusa	*An. vagus*	5	80	20	0	80	20	0

Tenggara	*An. subpictus*	5	100	0	0	100	0	0
	
	*An. sundaicus*	4	100	0	0	100	0	0

Aceh	*An. maculatus*	2	100	0	0	100	0	0

West Sulawesi	*An. barbirostris*	3	100	0	0	100	0	0
	
	*An. sulawesi*	1	100	0	0	100	0	0
	
	*An. peditaeniatus*	1	100	0	0	100	0	0
	
	*An. nigerrimus*	1	100	0	0	100	0	0

### Frequency distribution of the *rdl *allele

In this report, the frequency distribution of the *Rdl *allele in each species examined could not be determined as many of them were represented by an individual sample. In Central Java and Lampung Provinces, however, it was evident that *An.s vagus*, *An. aconitus *and *An.s barbirostris *represent the species with the higher *Rdl *mutant allele frequency, respectively. The three species are well known to closely associate with agriculture area as mostly use rice field or stream in inland area as their breeding sites.

## Discussion

The *Rdl *gene fragment encompassing the mutational sites associted with dieldrin resistance has been successfully amplified and sequenced in 19 species of Anopheline mosquitoes in indonesia and the DNA sequences have been deposited in the GenBank. Molecular analyses of the *Rdl *gene of the *Anopheles *malaria vectors collected in 10 endemic areas of Indonesia: Aceh, North Sumatra, Bangka Belitung, Lampung, Central Java, East Nusa Tenggara, West Nusa Tenggara, West Sulawesi, Molucca and North Molucca indicated a high sequence conservation at the protein levels to the previously published *Rdl *sequence [25]. The existence of the resistant *Rdl *allele in five provinces - the *Rdl *302S in four provinces: North Sumatra, Lampung, Central Java and West Nusa Tenggara, among *An. vagus, An. aconitus*, *An. barbirostris, An. sundaicus *and *An. nigerrimus *and the *Rdl*302G in the *An. farauti *in Molucca Province, was revealed. This finding indicates that cyclodiene insecticides pressures along this specific target in anopheline mosquitoes are still in place in many malaria-endemic areas of Indonesia. Cyclodiene insecticides were used in malaria control programmes during the 1955 following the spread of mosquito resistance to DDT, and so far, dieldrin was the only cyclodiene insecticide that had been used in the programme in Indonesia This insecticide was used only for a short period following the discovery of doubly-resistant *An*. *aconitus *to DDT and dieldrin in Central Java in 1965 [16]. However, in agricultural areas, several cyclodiene insecticides are currently still in use such as, endosulfan, aldrin and heptachlor, and resistance of these insecticides by various agricultural insects has been documented in several areas [28].

Although the resistence to dieldrin had been first documented in *An. aconitus*, in Central Java in 1965, this finding is the first report of the existence of the *Rdl *dieldrin-resistant alleles, A302A/G in Indonesian Anopheline vectors. As dieldrin is no longer used in Indonesia, the existence of *Rdl *mutant allele in many *Anopheles *species in Indonesia might be associated with either the use of cyclodiene insecticides in agriculture or the relative high fitness of the mutant alleles in comparison to the wildtype.

In conclusion, this study reports the the existence of the *Rdl *mutant alleles among the major malaria vectors in Indonesia and their existence might be associated with insecticide use in agricultural area. Further biochemical study to assess the sensitivity of the *Anopheles *that carries the *Rdl *allele to the cyclodiene insecticides used in agriculture are now in progress.

## Competing interests

The authors declare that they have no competing interests.

## Authors' contributions

DS, LS, PBSA, IEPR, NRP, SSM, S, WM and DSA performed molecular assays, data analysis, and the manuscript writing. PBSA and LS have equal contribution for this study. SS, S collected field samples and performed data analysis. DS, SS, FL, NFL, and WH designed the study and manuscripts writing were responsible for management and fund raising for this study. All authors read and approved the manuscript. This study is part of the thesis for Master of Science Programme at the University of Indonesia for LS.
